# Multidimensional Differences and Driving Mechanisms of Bacterial Communities in Urban and Rural Rivers Across China

**DOI:** 10.3390/microorganisms14061185

**Published:** 2026-05-24

**Authors:** Lina Wu, Shuai Lu, Fanjin Ye, Jinxia Lu, Xiaoling Liu, Yanfang Tian

**Affiliations:** 1 Laboratory of Urban Stormwater System and Water Environment (Ministry of Education), Beijing University of Civil Engineering and Architecture, Beijing 100044, China; wulina@bucea.edu.cn (L.W.); 2108570023005@stu.bucea.edu.cn (S.L.); 2 Research Center for Advanced Nitrogen and Phosphorus Removal with Desulfurization from High-Ammonia Nitrogen Wastewater, Institute of Advanced Materials, Beijing University of Civil Engineering and Architecture, Beijing 100044, China; 3 State Key Laboratory of Environmental Criteria and Risk Assessment, Chinese Research Academy of Environmental Science, Beijing 100012, China; yfj0609@163.com (F.Y.); lujx@craes.org.cn (J.L.); 4 State Environmental Protection Key Laboratory of Estuarine and Coastal Environment, Chinese Research Academy of Environmental Science, Beijing 100012, China

**Keywords:** planktonic bacteria, community structure, co-occurrence network analysis, functional prediction, community assembly

## Abstract

This study systematically compared the structural, functional, pathogenic, and assembly-mechanism characteristics of bacterial communities between urban and rural rivers across China, based on integrated water quality data from 421 sampling sites and 16S rRNA gene sequences from 475 sampling sites. The results revealed that urban rivers had significantly higher nutrient concentrations and bacterial α-diversity, along with lower β-diversity. Urban rivers were enriched with organic matter-degrading phyla such as Chloroflexi and Acidobacteriota and might exhibit more complex co-occurrence networks (average degree: 85.41). In contrast, rural rivers were enriched with phyla including Firmicutes and Cyanobacteria, as well as genera such as *Exiguobacterium* and *Limnohabitans*, and might display higher network modularity (modularity: 0.59) and greater spatial heterogeneity in community composition. Functional prediction indicated stronger carbon-cycling potential in urban rivers, whereas nitrogen-cycling functions did not differ between the two river types. Regarding pathogen composition, urban rivers contained a higher number of pathogen species than rural rivers. It was suggested that stochastic processes dominated community assembly in both systems; however, heterogeneous selection contributed more strongly in urban rivers (14.7%). Overall, this work elucidated systematic differences in bacterial community structure, function, pathogen profile, and assembly mechanisms between urban and rural rivers, offering a scientific foundation for differentiated watershed management.

## 1. Introduction

River ecosystems serve as a fundamental basis for safeguarding regional ecological security and achieving sustainable development. Their ecological health is directly linked to water quality and the integrity of biogeochemical cycling processes [1]. Within these ecosystems, planktonic bacteria represent a key biological component. They not only act as a core engine driving the cycling of essential biogenic elements such as carbon and nitrogen [2], but also are widely recognized as effective biological indicators for assessing aquatic ecological status and the degree of anthropogenic disturbance, owing to their high sensitivity and rapid response capability to environmental perturbations [3,4,5].

With the accelerating pace of global urbanization and agricultural intensification, human activities have become the dominant force altering river habitats and their associated microbial community structures. Urban rivers primarily receive point-source discharges such as industrial and domestic wastewater, often leading to nutrient enrichment, decreased dissolved oxygen (DO), and the input of toxic substances [6,7]. In contrast, rural rivers are more influenced by non-point source pollution from cultivation and aquaculture. Their pollutant inputs are characterized by dispersion, seasonality, and pronounced spatial heterogeneity [3,8]. This differential environmental pressure is theoretically expected to shape distinct microbial communities. However, existing research has predominantly focused on single river types or localized regions—for instance, studies confined to urban river sediment bacteria [9] or planktonic bacteria within specific rural watersheds [10]. A systematic, multidimensional, and integrated comparison of planktonic bacterial communities between urban and rural rivers on a large-scale is still lacking. Therefore, clarifying the overall differential patterns of bacterial communities between urban and rural rivers is crucial for revealing how anthropogenic disturbances reshape aquatic microbial ecology.

In recent years, the scope of microbial ecology research has continuously expanded, progressing from early descriptions of community composition to an integrated analysis of community assembly mechanisms, interspecies interaction networks, and ecosystem functions. Regarding mechanistic studies, analytical frameworks combining neutral models with phylogenetic null models can effectively quantify the contributions of stochastic and deterministic processes to community assembly and have been widely applied in ecosystems such as soils and wetlands [11,12]. Nevertheless, the systematic application of this framework in river ecosystems, particularly in comparative studies across urban and rural environments with significant environmental gradients, remains limited. This gap hinders a deeper understanding of the similarities and differences in community assembly mechanisms under these two habitat types. In terms of interspecies relationships, co-occurrence network analysis serves as a powerful tool for revealing microbial interaction patterns and identifying keystone species, and has seen progress in aquatic systems like lakes and reservoirs [13,14]. However, the topological structures and stability differences in microbial networks in urban versus rural rivers have not yet been clearly elucidated. On the functional front, function prediction based on databases (e.g., FAPROTAX) provides a feasible approach to link community structure with ecosystem function. Nevertheless, differences in the functional potential of bacterial communities between urban and rural rivers in key elemental cycles (e.g., carbon and nitrogen), as well as whether such differences correspond to their pollution characteristics, remain to be verified with large-scale datasets. Furthermore, anthropogenic pollution is frequently accompanied by the introduction of pathogenic microorganisms, thereby posing potential ecological and health risks [15]. However, differences in the composition and abundance of pathogens between urban and rural rivers have received relatively little attention.

Based on the above, this study compiled water quality data from 421 sampling sites and 16S rRNA gene sequences from 475 sites nationwide to systematically compare the planktonic bacterial communities between urban and rural rivers. By employing a comprehensive suite of methods, including diversity analysis, co-occurrence network construction, functional prediction, pathogen identification, and community assembly mechanism analysis, such as a neutral model and Infer Community Assembly Mechanisms by Phylogenetic-Bin-Based Null Model (iCAMP), this study aims to: (1) characterize the multidimensional differences in bacterial communities between urban and rural rivers; (2) and elucidate the dominant processes and potential environmental drivers underlying their community assembly mechanisms. These findings provide data support and a theoretical basis for further understanding the impacts of human activities on river microbial ecology, the implementation of differentiated watershed management, and the comprehensive assessment of aquatic ecosystem health.

## 2. Materials and Methods

### 2.1. Data Sources and Acquisition

This study compiled published water quality and microbial sequencing data via systematic literature retrieval and screening of river datasets. Literature search was primarily performed using the Web of Science Core Collection and China National Knowledge Infrastructure. The English search strategy was: TS = ((river OR stream) AND (bacteria* OR microbe) AND (community OR diversity OR structure) AND (16S* OR “high-throughput sequencing”) AND AD = (China)), excluding non-target habitats such as soil, marine, and gut environments. Chinese search was supplemented using keywords like “river”, “microbe”, “bacteria”, “community”. Initially, a total of 580 relevant studies were included. To ensure the comparability of water quality and microbial datasets from published studies, screening strategies were subsequently applied to reduce potential spatial and methodological heterogeneity, as described below: (1) Sampling sites could be clearly classified as urban or rural areas; (2) Studies that reported key water quality parameters or deposited raw sequencing data in the National Center for Biotechnology Information (NCBI), along with detailed primer information, were included; (3) For each water quality parameter, studies adopting identical or comparable analytical methods were preferentially retained; (4) For biological data, only datasets generated via Illumina paired-end sequencing platforms were selected to ensure consistent sequencing depth; (5) The hypervariable V4 region of the 16S rRNA gene (covering the V3–V4 and V4–V5 fragments) was targeted to guarantee adequate taxonomic resolution and reduce amplification bias [16]; (6) Samples collected between 2016 and 2025 were included to constrain the temporal scope and enhance comparability among datasets. Finally, a total of 36 eligible studies were retained for subsequent analyses, covering 421 water quality sampling sites and 475 microbial sampling sites [2,7,9,17,18,19,20,21,22,23,24,25,26,27,28,29,30,31,32,33,34,35,36,37,38,39,40,41,42,43,44,45,46,47,48,49] (Table S3). In accordance with the official classification framework issued by the State Council of the People’s Republic of China [50], all sampling sites were further classified into urban or rural categories.

Water quality data were extracted from 421 sampling sites (223 urban and 198 rural). Indicators with an occurrence frequency ≥30 were selected as key water quality parameters, including pH, DO, total organic carbon (TOC), chemical oxygen demand (COD), total phosphorus (TP), total nitrogen (TN), ammonium nitrogen (NH_4_^+^-N), nitrate nitrogen (NO_3_^−^-N), and nitrite nitrogen (NO_2_^−^-N). Their analysis methods and detection limits were summarized in Table S4. Values below the detection limit were excluded in subsequent analysis. Sequencing data were retrieved from NCBI using the SRA accession numbers reported in the published literature, and subsequently downloaded via the SRA Toolkit (v3.0.2). Raw sequence data were finally obtained from 475 samples (321 urban and 154 rural). Detailed information on biological data sources was provided in Table S4. Paired-end reads were merged and quality-filtered with a minimum quality threshold of 20. DADA2 was then used for denoising, chimera removal, and Amplicon Sequence Variants (ASVs) generation at 100% sequence similarity. Taxonomic annotation was performed based on the Silva database (v132) using the classify-sklearn plugin. Non-bacterial sequences were removed. Samples with fewer than 10,000 sequences and ASVs with fewer than 10 sequences were discarded. Rarefaction was performed to a uniform sequencing depth [51]. For subsequent analyses except pathogen identification, taxonomic assignments were aggregated at the genus level. Specifically, ASVs sharing the same full taxonomic lineage (from phylum to genus) were merged into a single genus-level feature, and their abundance sums were calculated accordingly.

### 2.2. Microbial Analysis

Bacterial community alpha diversity was calculated using Chao1, ACE, Shannon, and Pielou indices, with between group differences assessed for significance using nonparametric tests. Co-occurrence networks were constructed at the genus level using Spearman correlation (|r| > 0.5, *p* < 0.01), visualized and analyzed in Gephi (v0.10.1) to calculate topological parameters (average degree, modularity, clustering coefficient, etc.). Keystone species were identified based on Zi-Pi values (Zi > 2.5, Pi > 0.62). Differentially abundant taxa were screened using LEfSe analysis (LDA score > 3.5). Community assembly mechanisms were analyzed using both the Neutral Community Model (NCM) and null models: the NCM assessed the contribution of stochastic processes by fitting R^2^ and Nm; based on βNTI and RC indices calculated from a phylogenetic tree, the iCAMP model quantified the relative contributions of five ecological processes: dispersal limitation, homogeneous dispersal, homogeneous selection, heterogeneous selection, and undominated processes. Bacterial community functions were predicted using the FAPROTAX database. All analyses were performed in the R language environment (v4.3.1), primarily relying on packages such as vegan, microeco, Hmisc, minpack.lm, and igraph.

### 2.3. Pathogen Identification

According to the method of Yang et al. [52], all pathogen sequence files were downloaded from Microbial Pathogen Database (MBPD), covering a total of 72,685 sequences of animal, plant, and zoonotic pathogens. Local sequence alignments were performed using NCBI BLAST+ (v2.12.0, https://blast.ncbi.nlm.nih.gov (accessed on 1 January 2026)). For each ASV, alignments were conducted using thresholds of 95% similarity and 90% coverage. Only the best matching result was retained for each ASV, and species-level pathogenic data were compiled by consolidating matched results. Due to the limited taxonomic resolution of the 16S rRNA gene, identification is only reliable at the species level. It cannot differentiate between pathogenic and non-pathogenic strains, nor between obligate and opportunistic pathogens. Consequently, all identified pathogenic species were retained in this study. Since MBPD integrate multiple authoritative pathogen databases and exhibits high reliability and validity, cross-validation with other databases was therefore omitted.

## 3. Result and Analysis

### 3.1. Comparative Analysis of Water Quality Parameters

**Figure 1:**
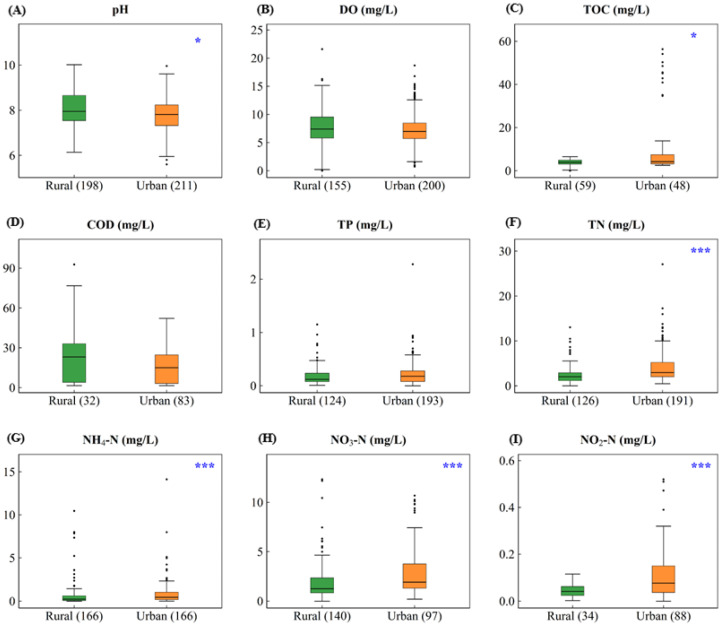
Comparison of key water quality parameters between urban and rural rivers. (**A**) pH; (**B**) DO; (**C**) TOC; (**D**) COD; (**E**) TP; (**F**) TN; (**G**) NH_4_-N; (**H**) NO_3_-N; (**I**) NO_2_-N. The numbers in parentheses indicate the sample size for each parameter and error bars represent standard deviation. Multiple comparisons are conducted using the Kruskal–Wallis test (* *p* < 0.05, *** *p* < 0.001).

Urban and rural rivers exhibited distinct differences in water quality parameters (Figure 1). Specifically, urban rivers showed significantly higher levels of organic nutrients and nitrogen: the average concentration of TOC reached 12.83 mg/L, significantly higher than the 3.70 mg/L in rural rivers (*p* < 0.05). The average concentrations of TN, NH_4_^+^-N, NO_3_^−^-N, and NO_2_^−^-N were 4.08 mg/L, 0.93 mg/L, 2.82 mg/L, and 0.120 mg/L, respectively, all significantly higher than those in rural rivers (2.62 mg/L, 0.62 mg/L, 1.93 mg/L, and 0.047 mg/L, respectively) (*p* < 0.001). The average pH was 7.75 in urban rivers, while it was 7.94 for rural rivers. No significant differences were found between the two river types concerning DO, TP, and COD, although rural rivers exhibited a wider fluctuation range in COD values.

### 3.2. Bacterial Community Composition and Pathogen Distribution

**Figure 2:**
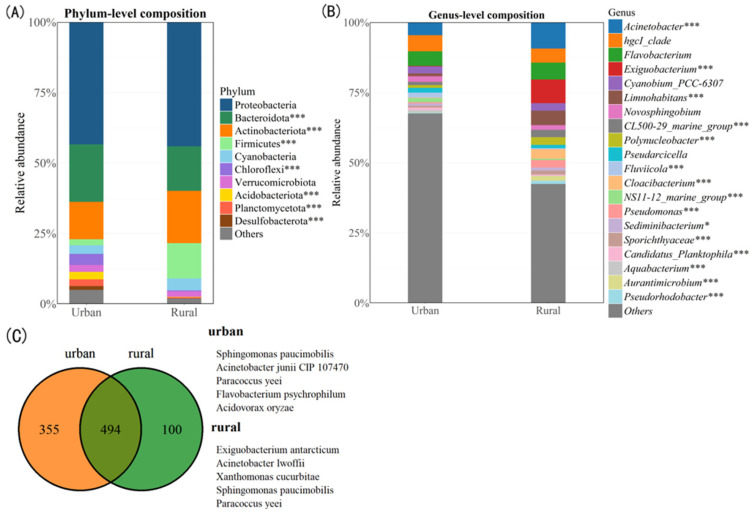
Differences in bacterial community composition between urban and rural rivers. (**A**) Relative abundance of the top 10 bacterial phyla; (**B**) Relative abundance of the top 10 bacterial genera; (**C**) Venn diagram showing the shared and unique pathogenic bacterial species between urban and rural rivers. Significance levels are denoted as follows: * *p* < 0.05, *** *p* < 0.001.

Significant differences in the bacterial community composition at both phylum and genus levels, as well as in pathogen profiles, were observed between urban and rural rivers (Figure 2). At the phylum level, Proteobacteria, Bacteroidota, and Actinobacteriota were the dominant phyla shared by both river types, though their relative abundances differed notably. In urban rivers, the dominant phyla were Proteobacteria (43.37%), Bacteroidota (20.38%), and Actinobacteriota (13.29%), with other phyla present at lower abundances. In rural rivers, the dominant phyla were similarly Proteobacteria (44.06%), Actinobacteriota (18.59%), and Bacteroidota (15.81%). It is noteworthy that the relative abundances of Firmicutes and Cyanobacteria were significantly higher in rural rivers compared with urban rivers (*p* < 0.05), whereas Chloroflexi and Acidobacteriota were significantly enriched in urban rivers (*p* < 0.05). At the genus level, *Flavobacterium*, *Acinetobacter*, and *hgcI_clade* were the shared dominant genera. Notably, the relative abundance of *Acinetobacter* was significantly higher in rural rivers (9.31%) than that in urban rivers (4.54%). Rural rivers were further characterized by *Exiguobacterium* and *Limnohabitans* as distinctive dominant genera.

**Figure 3:**
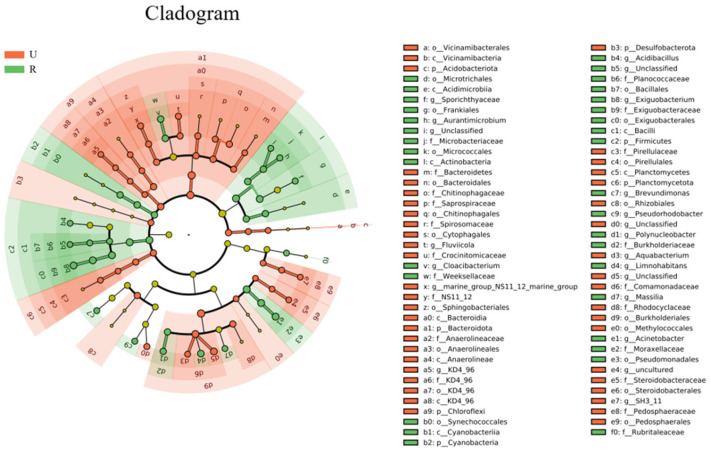
Differential indicator species between urban and rural rivers identified by LEfSe analysis. Linear Discriminant Analysis (LDA) score > 3.5. The cladogram shows taxa significantly enriched in urban or rural rivers from phylum to genus level.

LEfSe analysis further identified 77 differential indicator species (LDA > 3.5). The rural group was mainly enriched with taxa related to Firmicutes, Actinobacteriota, and Cyanobacteria, including genera such as *Exiguobacterium*, *Acinetobacter*, *Limnohabitans*, and *Cloacibacterium*. In contrast, the urban group was dominated by Chloroflexi, Acidobacteriota, Planctomycetota, and Desulfobacterota (Figure 3), with representative genera including *Aquabacterium*, *Chloroflexi KD4_96*, and *Pedosphaeraceae SH3_11*. Regarding pathogen composition, a total of 849 pathogen species were found in urban rivers and 594 in rural rivers, with 494 species shared between them (Figure 2C). Major pathogens in urban rivers included *Sphingomonas paucimobilis*, *Acinetobacter junii*, *Paracoccus yeei*, *Flavobacterium psychrophilum*, and *Acidovorax oryzae*; while rural rivers were dominated by pathogens such as *Exiguobacterium antarcticum*, *Acinetobacter lwoffii*, *Sphingomonas paucimobilis*, and *Paracoccus yeei*.

### 3.3. Alpha and Beta Diversity of Bacterial Communities

**Figure 4:**
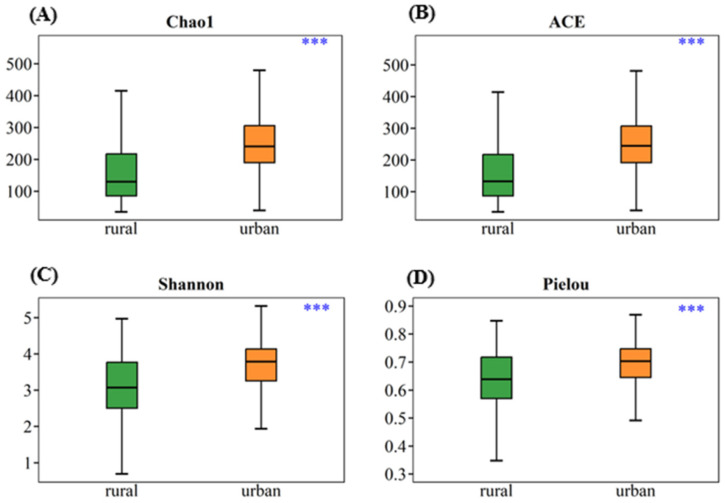
Comparison of bacterial community alpha diversity between urban and rural rivers. (**A**) Chao1 richness index; (**B**) ACE richness index; (**C**) Shannon diversity index; (**D**) Pielou evenness index. Multiple comparisons are conducted using the Kruskal–Wallis test (*** *p* < 0.001).

**Figure 5:**
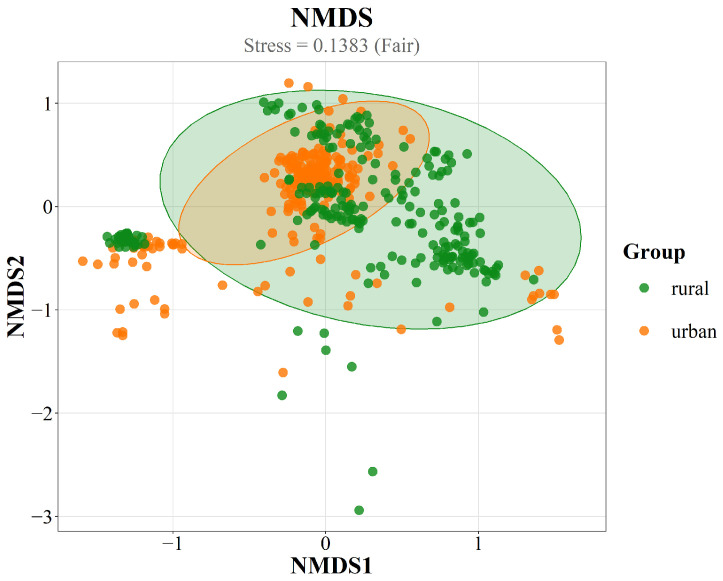
NMDS analysis of bacterial community beta diversity between urban and rural rivers. Ellipses represent 95% confidence intervals.

The α-diversity of bacterial communities in urban rivers was significantly higher than that in rural rivers (*p* < 0.01), and the community structures between the two groups were significantly different (Figure 4 and Figure 5). The mean values of Chao1, ACE, Shannon, and Pielou indices for urban rivers were 253.2, 253.8, 3.67, and 0.68, respectively, all significantly higher than the corresponding mean values of rural rivers (167.4, 168.4, 3.18, 0.64) (*p* < 0.01). Non-metric Multidimensional Scaling (NMDS) analysis further revealed that community composition among samples from rural rivers was more variable and relatively dispersed; whereas samples from urban rivers clustered more tightly together, indicating greater similarity in bacterial community structure across different sampling sites in urban rivers.

### 3.4. Co-Occurrence Network Structure and Keystone Species Identification

**Figure 6:**
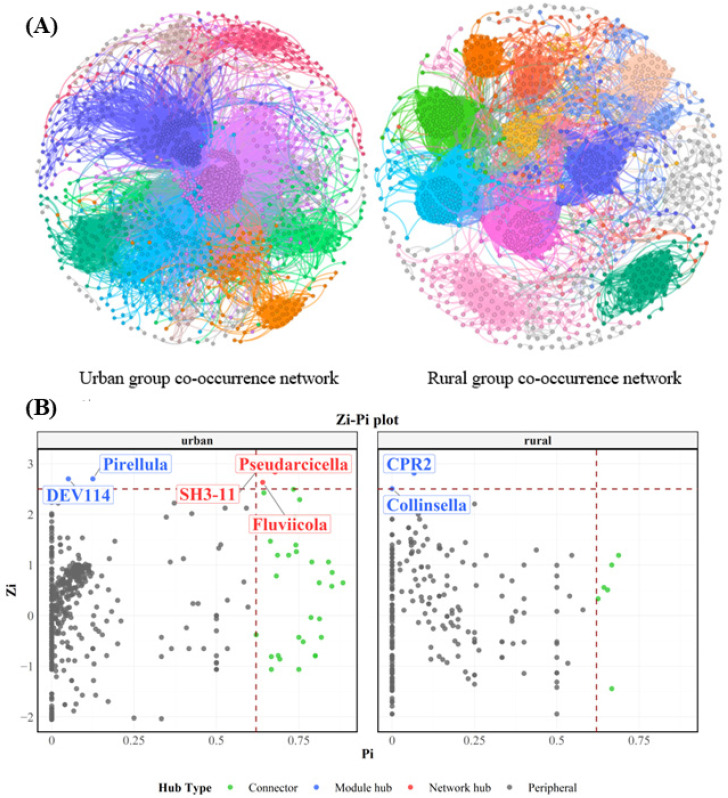
Co-occurrence network topology and keystone species of bacterial communities between urban and rural rivers. (**A**) Microbial co-occurrence networks constructed at the genus level (|r| > 0.6, *p* < 0.01). Node colors represent module affiliation, and node size is proportional to degree centrality; (**B**) Identification of keystone species based on Zi-Pi plot. Species are categorized into network hubs, module hubs, connectors, and peripherals according to thresholds (Zi = 2.5, Pi = 0.62).

The co-occurrence network structure and ecological functional characteristics of bacterial communities further revealed significant differentiation in microbial interaction patterns and ecosystem processes between urban and rural rivers. Network analysis indicated that bacterial communities of urban river possessed a more complex co-occurrence network (676 nodes and 28,868 edges) (Figure 6A). Its higher average degree (85.41), network density (0.13), and clustering coefficient (0.80) may reflect closer and more frequent interactions among microbial taxa. Keystone species such as *Pseudarcicella*, *SH3-11*, and *Fluviicola* occupied central positions in the network (Figure 6B), playing an important role in maintaining overall structural stability. In contrast, the rural river network exhibited higher modularity (0.59) and lower connectivity (average degree 15.74), indicating a community structure tending towards functional differentiation, with various groups operating within relatively independent modules. Only module hubs like *CPR2* and *Collinsella* were identified.

### 3.5. Prediction of Community Metabolic Functions

**Figure 7:**
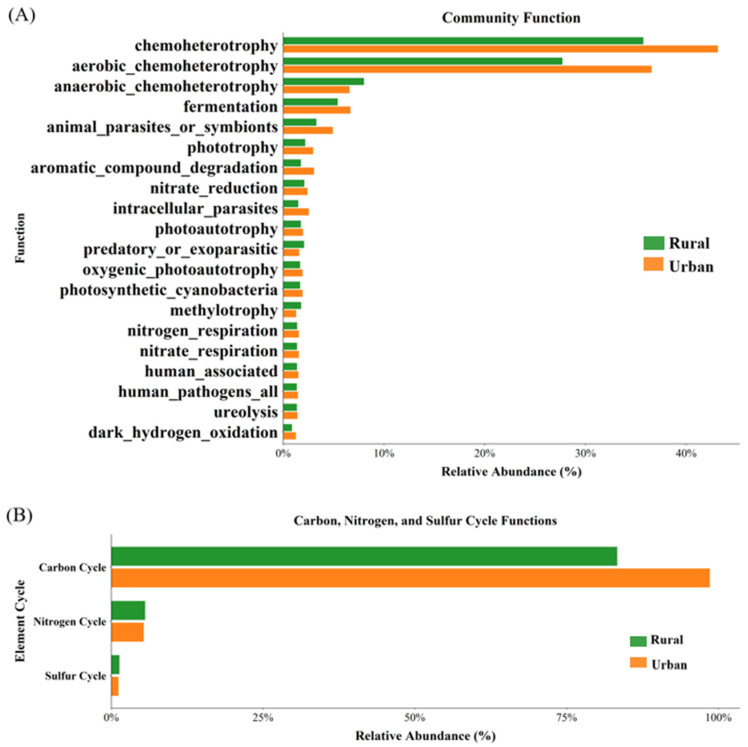
Functional prediction of bacterial communities between urban and rural rivers based on FAPROTAX. (**A**) Comparison of the top 20 most abundant predict metabolic functions; (**B**) Differences in the relative abundance of key functions involved in carbon, nitrogen, and sulfur cycles.

Functional prediction showed that chemoheterotrophy was the core metabolic function shared by both river types. (Figure 7A) However, urban rivers suggested a relatively higher potential for carbon cycle-related functions (e.g., fermentation, aromatic compound degradation), consistent with the degradation functions of enriched phyla like Chloroflexi and Bacteroidota. Although urban rivers had higher nitrogen nutrient concentrations, the predicted potential for nitrogen cycling functions in their bacterial communities showed no significant difference from rural rivers (Figure 7B). This may reflect divergence in nitrogen metabolic pathways: urban river communities might lean more towards oxidative processes like ammonification and nitrification, while taxa enriched in rural rivers, such as *Acinetobacter* and *Limnohabitans*, may hold a relative advantage in reductive or removal pathways like denitrification and nitrogen fixation, thus forming distinct nitrogen transformation patterns at the functional level.

### 3.6. Analysis of Community Assembly Processes

**Figure 8:**
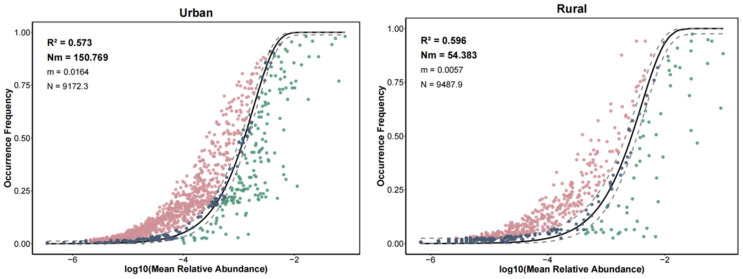
Fit of NCM to bacterial community assembly between urban and rural rivers. The solid line represents the model fit. R^2^ indicates the goodness of fit, and N_m_ represents the metacommunity size time migration rate.

**Figure 9:**
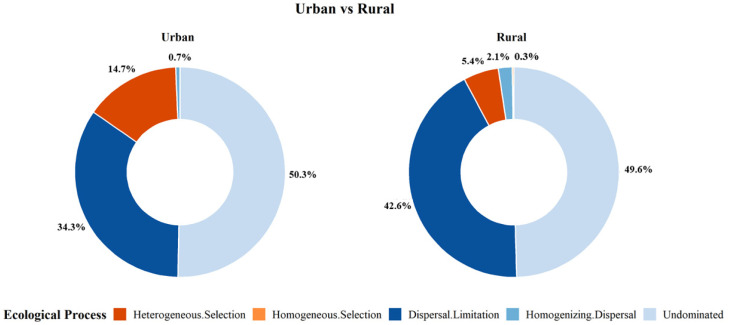
Quantification of bacterial community assembly processes between urban and rural rivers using the iCAMP model. The bar chart shows the relative percentage contributions of five ecological processes (heterogeneous selection, homogeneous selection, dispersal limitation, homogenizing dispersal, and undominated processes) to community assembly.

Community assembly is governed by five ecological processes as defined by iCAMP [11,53] specifically: (1) homogeneous selection, where consistent selective pressure under similar environmental conditions leads to community convergence; (2) heterogeneous selection, where divergent selective pressure under different environmental conditions leads to community divergence; (3) dispersal limitation, where low dispersal rates constrain species exchange; (4) homogenizing dispersal, where high dispersal rates promote community similarity; (5) un-dominated processes, where weak selection, weak dispersal, or ecological drift dominates community turnover. The assembly mechanisms of bacterial communities between urban and rural rivers were dominated by stochastic processes, yet the extent of influence from deterministic processes differed significantly (Figure 8 and Figure 9). Fitting based on the neutral model indicated that community assembly in these two rivers exhibited a reasonably high degree of explanation by neutral processes (urban: R^2^ = 0.573, Nm = 150.769; rural: R^2^ = 0.596, Nm = 54.383). Further quantification of the contributions of various ecological processes via null model analysis suggested that heterogeneous selection accounted for 14.7% in urban rivers, significantly higher than 5.4% in rural rivers (*p* < 0.05). In contrast, the proportion of dispersal limitation was greater in rural rivers, reaching 42.6%, compared with 34.3% in urban rivers. The remaining ecological processes, including homogeneous selection, homogeneous dispersal, and undominated processes, contributed relatively lower proportions.

## 4. Discussion

### 4.1. Water Quality Disparities Between Urban and Rural Rivers

The differences in water quality between urban and rural rivers are likely associated with fundamentally distinct pollution input patterns. Urban rivers are mainly influenced by high-intensity point source pollution, where industrial and domestic wastewater discharges lead to significantly higher concentrations of TOC, TN, and various nitrogen forms (such as NO_3_^−^-N and NO_2_^−^-N) compared with rural rivers (*p* < 0.05) [54]. In contrast, rural rivers are predominantly affected by agricultural non-point source pollution, with nutrients such as nitrogen and phosphorus originating from combined inputs of crop cultivation, animal husbandry, and domestic wastewater [8]. Although there is no significant difference in TP concentration between the two river types, studies suggest that the dominant forms of phosphorus may differ: urban rivers are primarily dominated by soluble reactive phosphorus; whereas rural rivers tend to be more influenced by particulate phosphorus [55]. In summary, these water quality differences constitute potential environmental drivers that may shape the distinct microbial community structures between urban and rural rivers.

### 4.2. Structural and Diversity Differences in Bacterial Communities Between Urban and Rural Rivers

Differences in the structural and diversity of bacterial communities between urban and rural rivers may systematically reflect the environmental selection pressures imposed by distinct anthropogenic disturbance patterns. The identification of 77 differential indicator species (LDA > 3.5) through LEfSe analysis further clarifies that urban and rural rivers are enriched with distinct microbial groups, and implied divergent pollution sources and ecological processes.

The bacterial communities in urban rivers appear to be associated with continuous point-source inputs, characterized by high nutrient levels. At the phylum level, the significant enrichment of Chloroflexi and Acidobacteriota may not only originate from wastewater treatment plant effluents and disturbed soil inputs but also associate with their key roles in degrading complex organic matter and driving carbon cycling [56]. Additionally, Planctomycetota and Desulfobacterota are commonly found in urban rivers. The former is closely linked to anthropogenically disturbed water bodies [54,57], while the latter participates in sulfide oxidation [58], together suggesting active heterotrophic metabolism and sulfur cycling processes in urban waters. At the genus level, *Aquabacterium* is commonly found in rivers receiving reclaimed water treatment plant effluents [49,59]; while the *KD4_96* group has been observed to enrich in rhizosphere environments under high pollution conditions [60]. The enrichment of these groups highlights the direct shaping of microbial composition by urban wastewater discharge and intense anthropogenic pollution.

In contrast, the structure of bacterial communities in rural rivers is more influenced by agricultural non-point source pollution and relatively natural hydrological conditions. The significant enrichment of Firmicutes and Actinobacteriota is a prominent feature. The former strongly indicates pollutant discharge from livestock manure or domestic wastewater [61]; while the latter exhibits higher activity under oligotrophic conditions and may possess a competitive advantage due to the relatively lower nutrient levels in rural rivers [62,63]. The increased proportion of Cyanobacteria suggests better light penetration and a potentially nitrogen-limited ecological state. Genera enriched in rural rivers, including *Exiguobacterium*, *Acinetobacter*, and *Limnohabitans*, are typically associated with agricultural runoff, crop rhizospheres, or oligotrophic freshwater conditions [36,59,64,65]. Meanwhile, the higher abundance of *Flavobacterium* in rural rivers aligns with its affinity for high DO and low ammonium conditions, as well as its adaptation to fishery-derived and domestic pollution inputs [10,66].

At the diversity level, these habitat differences directly lead to distinct diversity patterns. Urban rivers exhibit significantly higher α-diversity and more homogeneous community structures (lower β-diversity). This is primarily attributed to continuous and relatively homogeneous high nutrient inputs, which create rich ecological niches supporting the coexistence of more taxa [67]. Simultaneously, frequent hydrological disturbances promote microbial dispersal and weaken spatial isolation, resulting in higher and more convergent microbial diversity at the regional scale [68]. In contrast, the lower nutrient levels, more heterogeneous pollution inputs, and more pronounced natural hydrological gradients in rural rivers collectively contribute to lower bacterial diversity and more pronounced spatial differentiation [69,70]. This diversity difference may essentially illustrate the differential restructuring of natural microbial biogeographic patterns by intense, homogeneous anthropogenic disturbances versus dispersed, heterogeneous agricultural activities.

### 4.3. Co-Occurrence Network and Pathogen Differences Between Urban and Rural Rivers

Network analysis shows that urban river bacterial communities possess more complex co-occurrence networks. This could relate to the relatively homogeneous and resource-rich environment of urban waters, which supports broader niche overlap and competitive-cooperative relationships [71]. Additionally, the diverse functional zones within urban areas may contribute to more complex co-occurrence patterns and potentially influence network stability. In contrast, higher modularity and lower connectivity of rural river networks suggest that their community structure tends toward functional differentiation, with various taxa functioning within relatively independent modules [72]. Only a few module hubs, such as *CPR2* and *Collinsella*, were identified, which are closely associated with scattered pollution inputs and greater spatial heterogeneity in hydrological and nutrient conditions in rural river habitats [73]. It should be noted that the co-occurrence networks are constructed based on statistical correlations, which do not necessarily represent actual ecological interactions. Elucidating genuine biological interactions require further in-depth analysis.

Pathogen analysis further highlights differences in pollution sources. Urban rivers harbor more pathogen species (849 species), dominated by *Sphingomonas paucimobilis*, *Acinetobacter junii*, and others commonly found in sewage and human activity-intensive environments [74], suggesting that point-source discharges markedly elevate pathogen-related health risks in urban waters. Rural rivers harbor fewer total pathogens (594 species) yet are enriched in species associated with low-temperature environment or animal intestinal habitats, such as *Exiguobacterium antarcticum* and *Acinetobacter lwoffii*, suggesting that agricultural runoff, livestock farming, and cold habitats collectively shape their pathogen composition [75,76]. It should be noted that the number of detected pathogen species may show potential health risks, but their viability and infectivity require further confirmation through more precise experimental data.

These differences in network and functional characteristics collectively demonstrate that intense and homogeneous anthropogenic disturbance in urban rivers suggests highly interconnected bacterial communities with active carbon metabolisms and higher pathogen loads. In contrast, under heterogeneous non-point source influences, rural rivers may develop distinct microbial ecological patterns characterized by pronounced modularity, differentiated nitrogen transformation functions, and pathogen composition closely associated with environmental features.

### 4.4. Analysis of Assembly Mechanisms and Ecological Processes Between Urban and Rural Rivers

The assembly processes of bacterial communities in urban and rural rivers are both dominated by stochasticity, but the types of deterministic processes that play a leading role are fundamentally different. This may reflect the essential distinctions between the two habitat types in terms of anthropogenic disturbance patterns and natural attributes. By integrating neutral model and phylogenetic null model analyses, this study may confirm that stochastic diffusion and ecological drift serve as the foundational forces maintaining microbial diversity in both river types. However, quantitative analysis further reveals that deterministic processes may shape community structure in distinct ways.

In urban rivers, intense and continuous point-source pollution inputs, such as domestic and industrial wastewater discharges, can generate a relatively stable and homogeneous nutrient environment [77,78]. This condition is associated with a significantly higher contribution of heterogeneous selection observed in this study (14.7%, compared with 5.4% in rural rivers), implying that environmental filtering may play a key role in shaping microbial community assembly. This result aligns with the general characteristics of urban rivers, which typically exhibit high nutrient levels, low DO, and specific organic pollution profiles [79,80]. In other words, bacterial community structure in urban rivers is predominantly shaped by a consistent “environmental filter” that may be driven by anthropogenic pollution. In contrast, rural river habitats may exhibit higher spatial heterogeneity. Their pollution sources are dispersed (non-point agricultural sources), and hydrological conditions are more variable, which may impose stronger constraints on microbial dispersal [81]. Dispersal limitation may contribute more substantially to community assembly in rural rivers (42.6%, compared with 34.3% in urban rivers), highlighting that geographical distance and hydrological connectivity may impose stronger constraints on their community composition. This finding is consistent with the principle that dispersal limitation intensifies with increasing spatial scale [82,83], and partly explains the greater differentiation of microbial communities among rural sampling sites, as reflected by their higher β-diversity and stronger network modularity.

### 4.5. Limitations of Data Integration

This study conducted a meta-analysis of water quality and biological data (16S rRNA gene sequences) retrieved from multiple published studies. Quality control was strictly implemented for the integration of heterogeneous datasets; nevertheless, several inherent limitations remain in this meta-analysis, which require further discussion. Firstly, the original studies were conducted across inconsistent sampling seasons and hydrological conditions, which might influence microbial community structure and water quality in both rural and urban rivers. Secondly, due to potential publication bias, sampling sites could not be evenly distributed between rural and urban rivers, meaning that the findings of our study reflect potential inferences rather than causal relationships. Thirdly, the 16S rRNA hypervariable regions used for amplification in all samples were V3–V4 and V4–V5. The V3–V4 region offers superior amplification efficiency for dominant bacterial such as Firmicutes and Bacteroidetes, whereas the V4–V5 region is more suitable for the detection of Archaea [84,85]. These two V4 hypervariable regions are widely recognized as among the most robust and informative segments for bacterial diversity analysis [86,87]. Previous studies have demonstrated that sequencing data from these two regions are highly correlated and share strong similarity at the genus and higher taxonomic levers [84,88]. However, different primer sets using for amplification of V3–V4 and V4–V5 may result in variation in taxonomic coverage and resolution, making potential cross-study comparisons difficult, especially for Archaea. Finally, raw individual-level data from all included studies were not accessible, leading this study to rely heavily on secondary data.

## 5. Conclusions

This study systematically compared the differences in the composition, diversity, co-occurrence networks, functional potential, and assembly mechanisms of planktonic bacterial communities between urban and rural rivers in China by integrating nationwide water quality and microbial sequencing data. The main conclusions are as follows: (1) Urban rivers exhibited significantly higher nutrient loads and bacterial alpha diversity. Their communities were enriched with phyla such as Chloroflexi and Acidobacteriota, and their co-occurrence networks were more complex and tightly connected. In contrast, rural rivers were enriched with Firmicutes, Cyanobacteria, and some genera such as *Exiguobacterium* and *Limnohabitans*. Their communities showed higher network modularity and greater spatial heterogeneity. (2) In terms of functional aspects, urban river bacterial communities were predicted to possess a potentially higher capacity for carbon cycling; whereas nitrogen cycling functions showed no significant difference between urban and rural rivers, implying a divergence in elemental cycling pathways between the two systems. Urban rivers may also harbor a greater diversity of pathogenic bacteria than that in rural rivers. (3) Regarding community assembly mechanisms, stochastic processes dominated in both river types; however, the contribution of heterogeneous selection may be significantly stronger in urban rivers. Rural rivers were more influenced by dispersal limitation, highlighting the spatial heterogeneity of their habitats.

This study revealed macro-scale divergence patterns in bacterial communities between urban and rural rivers through data integration. However, limitations in data sources and integration methods, such as inconsistencies in sampling seasons, hydrological conditions, and analytical approaches, may affect the interpretation of some results. Future studies could further combine seasonal monitoring and meta-genomic sequencing to elucidate the metabolic functions and ecological processes of key taxa at higher spatiotemporal resolutions. Additionally, greater emphasis should be placed on tracing and assessing biological risk factors such as pathogenic bacteria to provide more direct microbiological evidence for water quality safety management.

## Data Availability

All biological sequence data used in this study are publicly available through the National Center for Biotechnology Information (https://pubmed.ncbi.nlm.nih.gov/ (accessed on 1 January 2026)). All relevant site numbers are provided. The assembled and processed datasets are available from the corresponding author upon reasonable request.

## Supplementary Materials

The following supporting information can be downloaded at: https://www.mdpi.com/article/10.3390/microorganisms14061185/s1, Table S1: The information of the relevant points involved in the analysis of biological sequences; Table S2: Water Quality Data Table; Table S3: Source of water quality and microbial data.xlsx; Table S4: Water Quality Index Measurement Method.xlsx.
